# Radical cure for *Plasmodium vivax* malaria after G6PD qualitative testing in four provinces in Cambodia, results from Phase I implementation

**DOI:** 10.1186/s12936-024-04884-4

**Published:** 2024-02-23

**Authors:** Dysoley Lek, Yu-Cheng Tsai, Jillian Hirano, Siv Sovannaroth, Voeurng Bunreth, Prak Vonn, Or Vannthen, Tol Bunkea, Top Samphornarann, Nguon Sokomar, Mak Sarath, Soy Ty Kheang, Evelyn Wong, Michelle K. Burbach, Jayme Hughes, Huy Rekol

**Affiliations:** 1 National Center for Parasitology, Entomology, and Malaria Control, Phnom Penh, Cambodia; 2 Clinton Health Access Initiative, Phnom Penh, Cambodia; 3 Provincial Health Department, Ministry of Health, Phnom Penh, Cambodia; 4 Cambodia Malaria Elimination Project, Phnom Penh, Cambodia; 5 Population Services International, Phnom Penh, Cambodia; 6 Center for Health and Social Development, Phnom Penh, Cambodia; 7 School of Public Health, National Institute of Public Health, Phnom Penh, Cambodia; 8 Partnership for Vivax Elimination, Phnom Penh, Cambodia

**Keywords:** Malaria, Radical cure, Cambodia, Plasmodium vivax, Glucose-6-phosphate dehydrogenase, 8-aminoquinoline

## Abstract

**Background:**

Cambodia aims to eliminate all forms of malaria by 2025. In 2020, 90% of all malaria cases were *Plasmodium vivax.* Thus, preventing *P*. *vivax* and relapse malaria is a top priority for elimination. 14-day primaquine, a World Health Organization-recommended radical cure treatment regimen, specifically targets dormant hypnozoites in the liver to prevent relapse. Cambodia introduced *P. vivax* radical cure with primaquine after glucose-6-phosphate dehydrogenase (G6PD) qualitative testing in 2019. This paper presents Cambodia’s radical cure Phase I implementation results and assesses the safety, effectiveness, and feasibility of the programme prior to nationwide scale up.

**Methods:**

Phase I implementation was carried out in 88 select health facilities (HFs) across four provinces. Males over 20kgs with confirmed *P. vivax* or mixed (*P. vivax* and *Plasmodium falciparum*) infections were enrolled. A descriptive analysis evaluated the following: successful referral to health facilities, G6PD testing results, and self-reported 14-day treatment adherence. *P. vivax* incidence was compared before and after radical cure rollout and a controlled interrupted time series analysis compared the estimated relapse rate between implementation and non-implementation provinces before and after radical cure.

**Results:**

In the 4 provinces from November 2019 to December 2020, 3,239 *P. vivax*/mixed infections were reported, 1,282 patients underwent G6PD deficiency testing, and 959 patients received radical cure, achieving 29.6% radical cure coverage among all *P. vivax*/mixed cases and 98.8% coverage among G6PD normal patients. Among those who initiated radical cure, 747 patients (78%) completed treatment. Six patients reported side effects. In implementation provinces, an average 31.8 relapse cases per month were estimated signaling a 90% (286 cases) reduction in relapse compared to what would be expected if radical cure was not implemented.

**Conclusions:**

*Plasmodium vivax* radical cure is a crucial tool for malaria elimination in Cambodia. The high coverage of radical cure initiation and adherence among G6PD normal patients demonstrated the high feasibility of providing radical cure at point of care in Cambodia. Incomplete referral from community to HFs and limited capacity of HF staff to conduct G6PD testing in high burden areas led to lower coverage of G6PD testing. Phase I implementation informed approaches to improve referral completion and patient adherence during the nationwide expansion of radical cure in 2021.

**Supplementary Information:**

The online version contains supplementary material available at 10.1186/s12936-024-04884-4.

## Background

Individuals infected with *Plasmodium vivax* may suffer from relapses caused by dormant liver parasites (hypnozoites). Globally between 60 to 96% of *P. vivax* infections are due to hypnozoite reactivation [1–3]. These relapses can occur within variable timeframes post primary infection, ranging from less than one month to one year, which may also vary by geography [4]. Primaquine therapy, known as radical cure, is a World Health Organization (WHO)-recommended regimen for eradicating the liver stage of *P. vivax* malaria*.* However, there is a risk of potentially fatal haemolysis (acute haemolytic anaemia, or AHA) among G6PD deficient individuals, limiting the proportion of the population that can safely receive radical cure treatment with primaquine [5–7]. Therefore, WHO guidelines in 2016 recommend a 14-day primaquine regimen with 0.25 to 0.5 mg base per kilogram for patients without G6PD deficiency (“G6PD normal”), and an 8-week primaquine regimen with 0.75 mg base per kilogram for G6PD deficient patients when administered under medical supervision [8]. Additionally, the WHO recommends that patients’ G6PD status should be tested before prescribing primaquine [9].

Cambodia aims to eliminate all forms of malaria by 2025. In pursuit of this goal, the National Center for Parasitology, Entomology and Malaria (CNM) has led intensive efforts to reduce both *Plasmodium falciparum* and *P. vivax* cases in the country. Cambodia has made significant progress toward malaria elimination with a 71% decrease in total cases from 2019 to 2020. *Plasmodium falciparum* cases have dramatically decreased in recent years because of targeted malaria elimination interventions, leading to a rise in the proportion of *P. vivax* cases, from 84% in 2019, 90% in 2020 and 93% in 2021. In 2019, there were 8,298 *P. vivax* cases (90% of the national malaria burden) reported at an incidence rate of 1.65 cases per 1,000 population [10]. According to Cambodia’s 2014 treatment guidelines, *P. vivax* cases receive 3-day artesunate-mefloquine (ASMQ), the artemisinin-based combination therapy (ACT) first-line treatment for all malaria species, and 14-day primaquine (0.25–0.5 mg/kg/day) as radical cure if tested G6PD normal (deficiency defined as < 30% G6PD activity in males). An estimated 8–15% of the Cambodian population is G6PD deficient [11], posing a high risk of G6PD-deficient patients being prescribed primaquine. Therefore, including point-of-care testing for G6PD deficiency was a critical component in the phase I implementation design.

In November 2019, CNM launched the first phase of *P. vivax* radical cure in 88 health facilities (HFs) in four provinces, Battambang, Kampong Chhnang, Kampong Speu and Pailin, using CareStart rapid qualitative G6PD tests and providing 14-day primaquine treatment to eligible patients. An evaluation of the phase I implementation was conducted in December 2020 to assess its successes and challenges, and to inform the scaling up of radical cure at the national level in 2021.

## Methods

### Site selection

**Fig. 1:**
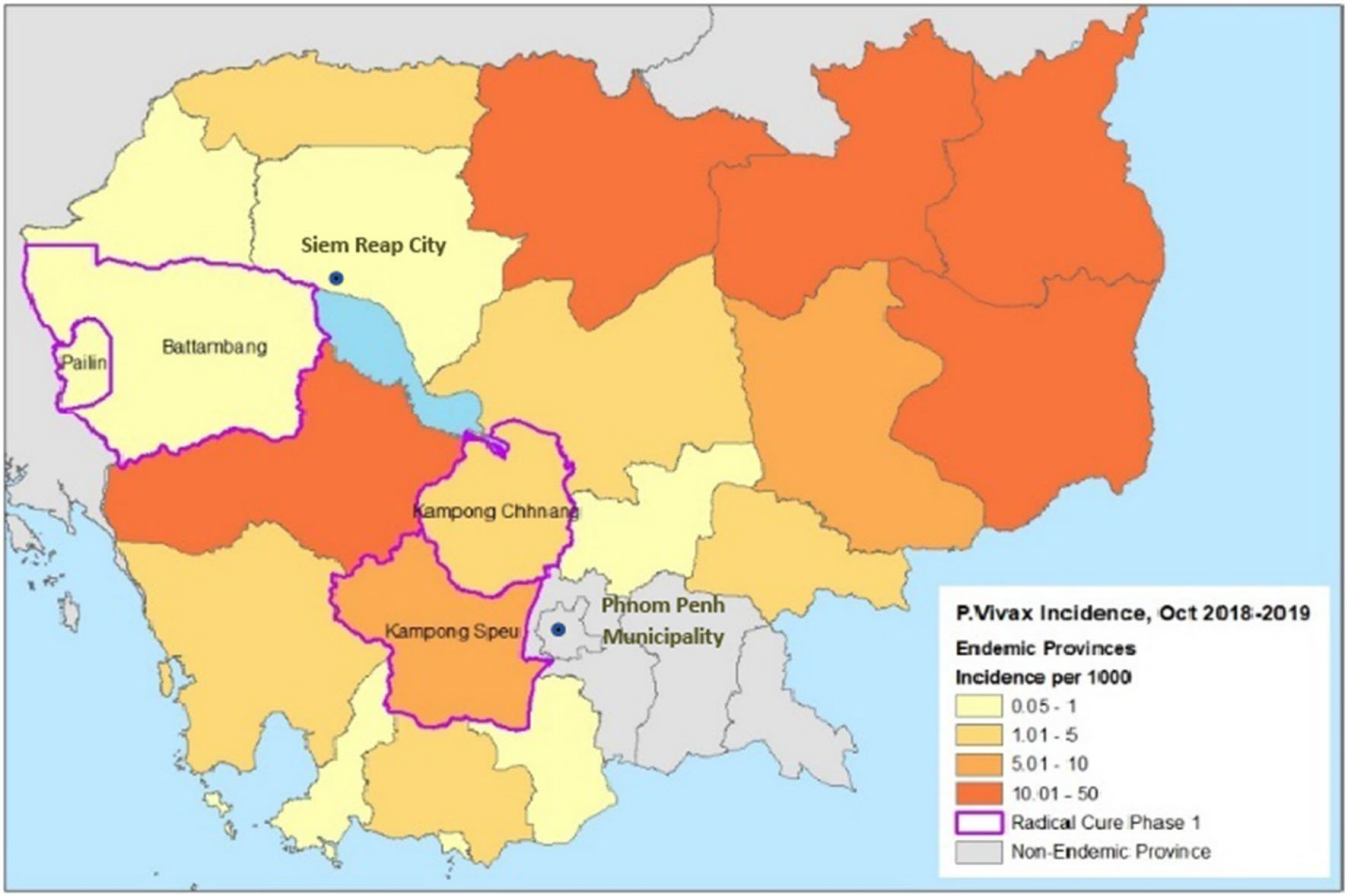
*Plasmodium vivax* incidence by province, Oct 2018–2019. Radical Cure provinces are outlined in purple

Among 21 malaria endemic provinces in Cambodia, four provinces were selected (Fig. 1) for phase I implementation of *P. vivax* radical cure. The provinces were selected as they contained geographies with both high and low case burden, high rates of G6PD deficiency, and close geographic distance to national hospitals.

HFs, which include health centres (HCs), district referral hospitals (RHs) and provincial hospitals (PHs), with an average of one or more *P. vivax* cases per month in 2018–2019 were selected for phase I. A total of 88 HFs were included: 76 health centres, 8 district referral hospitals, and 4 provincial hospitals.

### Implementation design

Phase I of *P. vivax* radical cure was implemented for 14 months from November 2019 to December 2020. In September 2019, following site selection, CNM conducted trainings on testing and treatment protocols, data collection and reporting, and monitoring adverse events to selected HFs. Cold chain supplies of refrigerators and cooler boxes were distributed to HFs during September and October to accommodate the required storage and usage temperature for G6PD tests between 18 and 32 °C [12], which is necessary given Cambodia’s climate.

### Testing for G6PD deficiency

In phase I, qualitative G6PD tests were used on *P. vivax* patients to determine their eligibility for treatment. At the time of radical cure introduction in 2019 the only G6PD rapid diagnostic tests available on the Global Fund External Review Panel list for procurement in Cambodia was CareStart qualitative G6PD rapid diagnostic tests. An important limitation of the qualitative G6PD tests is that they can only be used for males since the test is designed to distinguish G6PD deficiency, which is usually defined as 30% of the normal activity. G6PD deficiency is an X-chromosome linked disorder. As such, females can have “intermediate” G6PD deficiencies, rather than a binary outcome, which cannot be detected by the qualitative G6PD tests. As qualitative tests cannot provide definitive results for G6PD deficiency in females, women were ineligible to receive G6PD qualitative tests, and only male patients over 20 kg were included in phase I implementation. Children less than 20 kg were not tested with G6PD given that there was no paediatric dosage available.

In 2019, 85% of P. *vivax* cases in Cambodia occurred among males over 4 years old, indicating most at-risk patients would be eligible for phase 1 implementation [10]. Quantitative tests were used later during the nationwide scale up in 2021, allowing for both male and female *P. vivax*/mix patients to undergo G6PD testing. All male patients over 20 kg that tested positive for *P. vivax* infections were given 3 days ACT and a G6PD qualitative test at HFs. Cases detected by village malaria workers (VMWs), or mobile malaria workers (MMW) were provided ACT and referred to HFs for G6PD testing. G6PD testing was only conducted at HFs due to concerns about VMW/MMW capability to accurately interpret and store analyzers at the village level. Prior to administering G6PD testing and primaquine treatment, health providers at the HF provided patients a form describing the benefits and risks of radical cure and received informed consent to continue. Patients could choose to opt out, still receiving ACT.

### Treatment

In accordance with the 2019 WHO recommendations for preventing relapse in patients with *P. vivax* malaria, G6PD non-deficient males over 20kgs were given 14 days of primaquine (0.25–0.5 mg per kg per day according to dosing tables in Additional file 1: Table S1) and treatment counselling. They were followed up for self-reported adherence and side effects by HF staff via phone calls on days 3, 7 and 14 after treatment initiation. Patients with severe symptoms, including pallor, shortness of breath, palpitations, tachycardia, back pain or blood in the urine were referred to provincial hospitals for examination, and investigated by the Department of Drugs and Food (DDF) via the Adverse Drug Reaction (ADR) form for determining cause and strengthening pharmacovigilance in the country.

Primaquine was not given to G6PD deficient males who tested positive for *P. vivax* infection. These men were given ACT only and educated on recurring malaria, malaria preventative measures, and the importance of malaria testing when they have a fever. Ideally, G6PD deficient patients would receive 8-week primaquine treatment, however additional evidence on the feasibility, safety, and effectiveness of this treatment regimen in Cambodia is needed before nationwide scale up.

### Data collection

An electronic database managed by CNM, the Malaria Information System (MIS), includes nationwide patient-level malaria testing and treatment data, recorded by HFs and VMWs in real time. Given that radical cure was in the first phase of introduction, a separate paper register was initially used to record patient data, however later during scale up data was entered directly into MIS. Patient records in Phase I, including G6PD testing and radical cure treatment data, were recorded onto the paper registers kept at the HFs. HF staff recorded patient-reported adherence to treatment via phone calls on days 3, 7 and 14 of treatment, and these data were recorded in the paper register. Data from HFs’ paper registers were collected every month by civil society organization (CSOs) partners based in each province, aggregated by HF level, and shared with CNM to review during a monthly partner meeting.

In-depth key informant interviews were also conducted to collect provider feedback from health centre staff in implementation provinces. Interview questions covered aspects of patient and health centre staff experiences with radical cure treatment to gain a comprehensive understanding of the operational challenges of implementing radical cure protocols in the Cambodian context. All participants were informed that key informant interviews were voluntary, that responses would be anonymized, and that refusal to respond to any question at any point in the interview was permitted.

A total of 15 key informant interviews were conducted in July 2020 at facilities sampled to include a range of *P. vivax* burden (Additional file 1: Table S2). Among the participants that disclosed personal information, providers at radical cure facilities ranged in age from 28 to 56 and years of experience ranged from 3 to 30. Facilities sampled for key informant interviews had a range of 1–4 staff members dedicated to malaria work among facilities where participants disclosed the number of malaria staff. All interviews were conducted using a structured template and notes were recorded in Khmer, then later translated to English before undergoing analysis.

### Data analysis

A descriptive analysis for testing and treatment coverage of *P. vivax* radical cure was conducted. Key indicators include the number of *P. vivax* cases, *P. vivax* annual parasite incidence (API), VMW referral, G6PD testing, 14-day self-reported adherence and adverse events (Table 1).

**Table 1:** The indicators and their definition for the coverage of radical cure

Indicator	Definition
*P. vivax*/mixed cases	Number of all *P. vivax* or mixed (*P. falciparum* and *P. vivax*) cases confirmed positive by RDT or Microscopy during the study period
*Eligible P. vivax*/mixed cases	Number of *P. vivax*/mixed cases that were male patients over 20 Kgs among all *P. vivax*/mixed cases
*P. vivax* annual parasite incidence (API)	Number of confirmed new *P. vivax*/mixed cases expressed per 1,000 individuals in the population during the study period
VMW referral rate	Number of successful referral cases from VMWs (and MMWs) to HFs among all *P. vivax* eligible *P. vivax*/mixed cases detected by VMW/MMW and (e.g., males weighing or equal to 20 kg)
G6PD testing result	Number of G6PD normal and G6PD deficient patients, among all patients tested with G6PD test
14-day self-reported adherence	Number of cases reporting completion of 14-day treatment among all initiated radical cure
Adverse event	Number of cases reporting any adverse event symptoms among all cases received radical cure

Transcripts from the key informant interviews of health facility staff underwent an iterative thematic analysis compiling interviewee’s perspectives on the perceived patient experiences (i.e., patient counseling, referral, and side effects) and the provider experience (i.e., training, supervision, and stock outs).

To analyse the impact of the rollout of radical cure on *P. vivax* relapse cases, a controlled interrupted time series analysis was conducted. Informed by a review of the available literature, a relapse period of 30–274 days (one to nine months) was agreed upon [4, 13]. If a matched patient had received a prior diagnosis within the relapse period, then they were considered a relapse case. Matches with a difference of 30 days or fewer were not considered as relapse cases to account for potential treatment failure or duplicate records and consequently were excluded from the analysis.

Line lists of patient records were compiled from January 2018 to December 2020 in all intervention provinces and in five control provinces (no radical cure). A period of 12 months before and 14 months after implementation was examined. Kratie, Siem Reap, Kampong Thom and Oddar Meanchey were selected as the control provinces due to comparable baseline characteristics with the four pilot provinces. However, intervention provinces still represented a large burden of *P. vivax* in the country and, therefore, incidence and relapse rates in control provinces were lower than intervention provinces prior to implementation. A comparison of key epidemiological data from the intervention and control provinces is included in Additional file 1: Table S3. To link records by patient, a Jaro-Winkler (JW) similarity score was calculated from key patient identifying fields (name, sex, age and village of residence). If the JW score was less than 0.2 then the records were deemed to be from the same individual. A generalized least squares (GLS) model that included independently assessed autocorrelation terms was used to estimate the difference in post-intervention change in level of relapse cases in the intervention provinces, as well as the difference in post-intervention change in monthly relapse trends in intervention provinces, relative to controls.

Lag terms were identified through a Durbin Watson test and the examination of partial autocorrelation plots. Results of the GLS model were plotted using fitted line segments to illustrate pre- and post- intervention changes in level and trend of relapse cases (Additional file 1: Table S4). The change in the number of relapse cases and proportion of relapse cases, as well as the final proportion of relapse cases at the end of the intervention period were compared both between intervention and control provinces, and between the recorded results and the counterfactual. Data analysis was conducted using STATA (Release 16, StataCorp LLC, College Station, TX, USA) and R Studio. (v 4.0.2, Vienna, Austria). This paper analysed routine malaria programme data made available through the Cambodia MIS and paper registers entered into MIS by CNM. Authors had access to anonymous patient data only. The analyses were conducted according to CNM governmental guidelines and Clinton Health Access Initiative Study for Ethical Review Center (SERC).

## Results

**Fig. 2:**
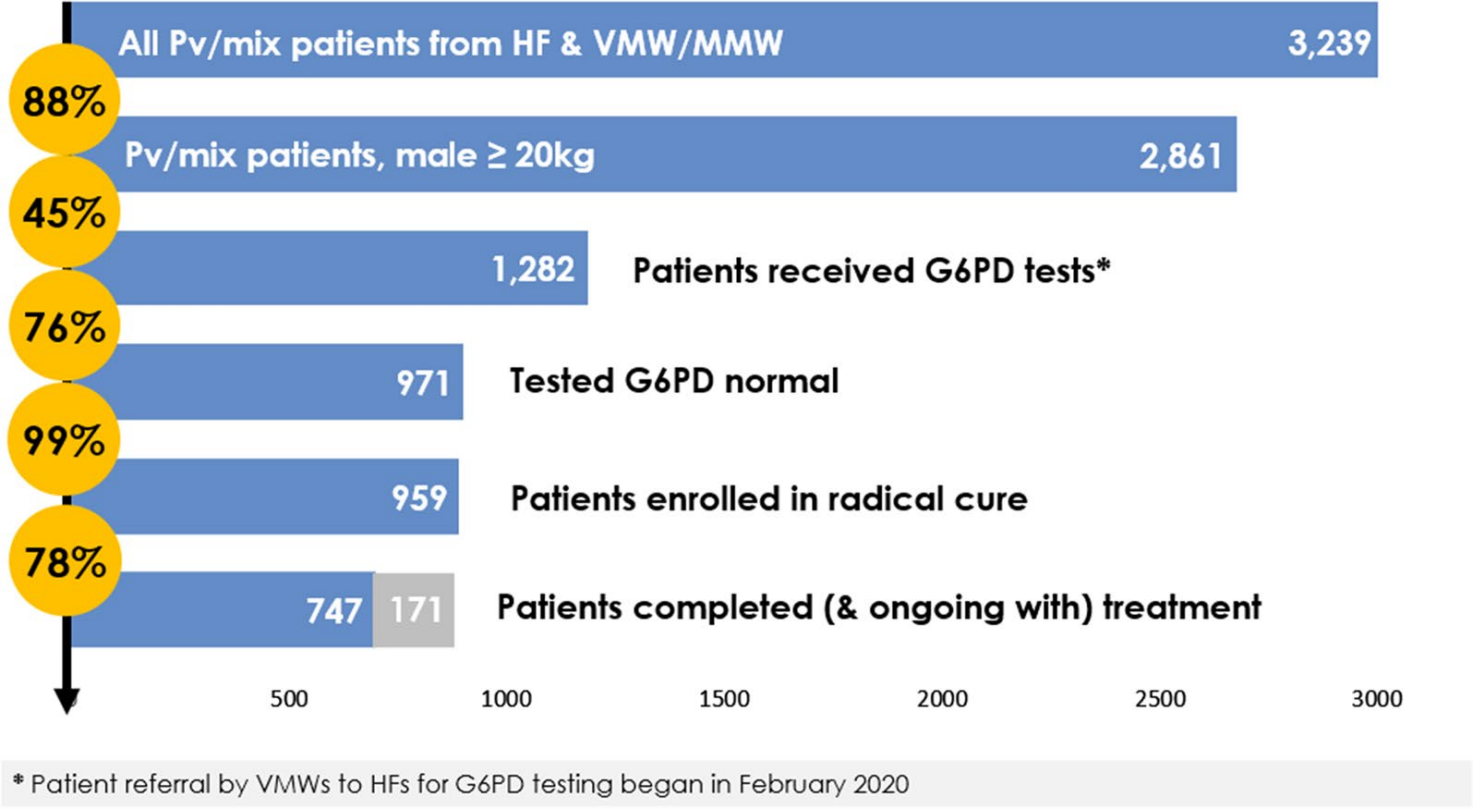
*Plasmodium vivax* cases diagnosed, G6PD tested, G6PD eligible, enrolled in radical cure, and completed treatment, Nov 2019-Dec 2020

In the 14-month pilot implementation from November 2019 to December 2020, 3,239 *P. vivax* infections were diagnosed, 1,282 patients received a G6PD test, and 959 G6PD normal patients received radical cure. Among all *P. vivax* patients, 29.6% received radical cure and among the *P. vivax* patients eligible for G6PD testing, 45% received a G6PD test (Fig. 2). Among the patients that received a G6PD test and tested G6PD normal, 99% were enrolled in radical cure for treatment, with 78% completing treatment at the time of the analysis period. At the provincial level, those with the higher number of cases (i.e., Kampong Chhnang and Kampong Speu) tended to have a fewer proportion of patients receive a G6PD test and complete 14-day primaquine regimen. This may be due to an overburdened health facility, competing priorities, and less time for HF staff to conduct patient follow up (Table 2).

**Table 2:** Number and percentages of patients in phase I provinces in each stage of diagnosis and treatment by operational districts (ODs), Nov 2019–Dec 2020

Provinces	Operational districts	Number of HFs	Pv cases	Pv cases eligible for G6PD test		Pv cases received G6PD test		Pv cases tested G6PD normal among all tested		Pv cases received radical cure among all G6PD normal cases		Pv cases completed & on-going for 14-day regimen among all received radical cure		Pv cases recorded adverse events among all received radical cure	
				n	%	n	%	n	%	n	%	n	%	n	%
**Battambang**	Battambang	9	66	49	(74%)	48	(98%)	43	(90%)	42	(98%)	41	(98%)	0	(0%)
	Maung Russei	6	61	49	(80%)	48	(98%)	45	(94%)	43	(96%)	42	(98%)	0	(0%)
	Sampov Luon	11	24	15	(63%)	14	(93%)	10	(71%)	9	(90%)	9	(100%)	0	(0%)
	Thma Koul	3	8	6	(75%)	6	(100%)	6	(100%)	6	(100%)	6	(100%)	0	(0%)
Summary for Battambang		29	159	119	(75%)	116	(97%)	104	(90%)	100	(96%)	98	(98%)	0	(0%)
**Kampong Chhnang**	Boribo	7	251	227	(90%)	132	(58%)	107	(81%)	107	(100%)	104	(97%)	2	(2%)
	Kampong Chhnang	8	282	261	(93%)	162	(62%)	132	(81%)	132	(100%)	131	(99%)	0	(0%)
	Kampong Tralach	6	78	72	(92%)	39	(54%)	37	(95%)	37	(100%)	37	(100%)	0	(0%)
Summary for Kampong Chhnang		21	611	560	(92%)	333	(59%)	276	(83%)	276	(100%)	272	(99%)	2	(1%)
**Kampong Speu**	Kampong Speu	7	1506	1303	(87%)	420	(32%)	281	(67%)	277	(99%)	252	(91%)	4	(1%)
	Kong Pisey	15	65	63	(97%)	43	(68%)	31	(72%)	31	(100%)	30	(97%)	0	(0%)
	Ou Dongk	8	377	344	(91%)	182	(53%)	134	(74%)	132	(99%)	128	(97%)	0	(0%)
	Phnom Srouch	6	505	457	(90%)	174	(38%)	132	(76%)	130	(98%)	125	(96%)	0	(0%)
Summary for Kampong Speu		36	2453	2167	(88%)	819	(38%)	578	(71%)	570	(99%)	535	(94%)	4	(1%)
**Pailin**	Pailin	5	16	15	(94%)	14	(93%)	13	(93%)	13	(100%)	13	(100%)	0	(0%)
Summary for Pailin		5	16	15	(94%)	14	(93%)	13	(93%)	13	(100%)	13	(100%)	0	(0%)
Total		88	3239	2861	(88%)	1282	(45%)	971	(76%)	959	(99%)	918	(96%)	6	(6%)

### Referral to HFs from VMWs/MMWs

**Fig. 3:**
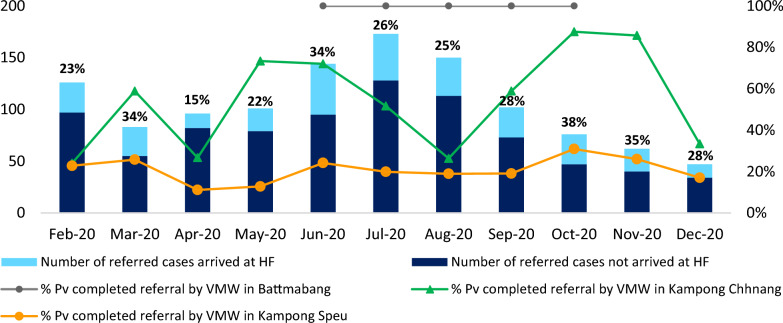
VMW referral rates for all pilot provinces, Feb 2020-Dec 2020

VMW/MMW referral was not in the pilot’s guidelines until early 2020 and this metric started to be tracked from February 2020. Among all *P. vivax* cases identified by VMWs, 27.3% completed referral to the nearest HFs for G6PD testing from February 2020 to December 2020 with a monthly referral rate ranging from 15 to 38%. A high variation in referral rate was also shown across provinces, with 100% in Battambang, 54% in Kampong Chhnang and 21% in Kampong Speu. Of note, Palin had no cases detected by VMW/MMW throughout this period, and Battambang only had referred cases for some months, resulting in limited or no data on VMW/MMW referral rate for these provinces (Fig. 3). Regardless of the fluctuation of the referral rate across months, the overall trend over time shows an increase in successful referral.

According to key informant interviews, travel distance to health facilities and limited transportation options were cited as a challenge, both for completing referrals to the health facility for G6PD testing and for conducting patient follow-up during the treatment period. Referrals for G6PD testing were reported as successful in instances where communication mechanisms and cooperation between the health centre, VMWs, and patients were strong. Specifically, referrals were reported as successful when VMWs called HCs in advance, village leaders were cooperative in the process, HC staff met with VMWs regularly and had established cooperative relationships at VMW monthly meeting, VMWs accompany patients to the HC for testing, HCs call VMWs to notify of completed referrals, and VMWs follow up via phone with patients who do not complete referral. VMW referrals were reported as unsuccessful where referred patients were mobile migrants, travel distance was far for the patient, and patients were sent by the VMW with just a treatment form and no prior HC communication. Referral is necessary given that HFs were the only place patients could receive G6PD testing and therefore radical cure treatment. Furthermore, 99% of patients that tested G6PD normal at HFs were enrolled in radical cure, making successful referral to HFs a very influential factor in achieving high coverage of radical cure.

### G6PD test results and interpretation

Among all the patients tested with G6PD qualitative tests (n = 1282), 76% (n = 971) tested G6PD normal and 24% (n = 311) G6PD deficient. An additional 127 tests were recorded as invalid, comprised of tests which produced unclear or illegible results or those discarded due to improper storage. Patients who received an invalid test were re-tested to identify their G6PD deficiency status. The distribution of G6PD results in phase I areas does not vary widely over time, but the percentage of G6PD normal test results in Kampong Speu was consistently lower than other phase I provinces, which might be explained by the fact that the province had the highest overall *P. vivax* cases in phase I areas. Possible reasons for incorrect G6PD results, which may cause lower average proportion of G6PD normal patients, are low quality and storage of G6PD RDTs, capacity of HF staff to conduct and interpret the tests, and the differences in population characteristics across provinces.

According to key informant interviews, the learning curve for conducting and interpreting G6PD tests was cited by some providers as a challenge, particularly in the first four months, which led to using extra test kits and extra blood draws for patients. Among staff with backgrounds in laboratory science, administration of G6PD tests was reported as less challenging. Additional support from CSOs and CNM at the health facility in the first months of implementation to review testing administration protocols were widely cited as helpful in alleviating these challenges.

### Patient enrollment in radical cure

Though G6PD testing did not capture many eligible patients, the radical cure enrollment rate among patients who received a G6PD normal result was extremely high (99%). Among providers interviewed, there were no reported instances of patients who were eligible for radical cure refusing enrollment in treatment. Eligible patients who had experienced prior relapses were particularly enthusiastic about enrollment in radical cure. Conversely, patients who were identified with a G6PD deficiency may not have fully understood the risks of primaquine treatment, expressing frustration to providers that they could not participate in radical cure. High enrollment rates among referrals also contributed to this effect, as patients travelling long distances to health facilities for G6PD testing are very likely to enroll in radical cure treatment upon receipt of G6PD normal test results.

### Follow up and adherence to treatment

Among the 959 patients enrolled in radical cure treatment, a total of 747 (77.9%) reported completing the full 14-day treatment regimen. An additional, 171 patients (17.8%) were categorized as treatment ongoing due to the data collection limitation that patients in the middle of the treatment at the end of the month when the data was collected were unable to confirm their completeness. 41 cases (4.3%) were reported as non-adherent during routine follow up, including six cases with adverse events that discontinued primaquine treatment (0.63%). Excluding the patients completing ongoing treatment, the completeness rate of 14-day treatment was 96% in the pilot implementation, and it did not vary widely across provinces or over time.

Additionally, patient follow-up was conducted mostly by phone (on days 3, 7 and 14 of treatment, though some facilities called patients as much as twice per day), but in-person follow up visits were completed in some areas. Caseload varied significantly between provinces, and consequently some areas had greater capacity to validate patient completion rates. In Battambang and Pailin, fewer *P. vivax* cases to manage allowed the CSO partners to accompany the HC staff to call or visit every patient, while in Kampong Speu, HC staff had higher caseloads to manage and CSOs also had less time to verify that HC staff completed each follow-up call.

Challenges to conducting follow up for treatment adherence were reported as part of the key informant interviews. Poor cellular coverage and unreachable patients were frequently mentioned, and secondary follow-up procedures varied by facility. In instances where patients were unreachable, health facilities had a wide range of responses, including assuming the patient was fine, reaching out to village leaders or family members to discuss the patient’s condition, or an in-person follow-up visit from a VMW or HC staff. Loss to follow up was often an issue for patients who are forest goers, frequently unreachable and/or did not have a mobile phone. Distance to the health facility was cited as a challenge to follow up, with some patients opting to stay with relatives closer to the health facility for the duration of the treatment period as their home village was too far and difficult to travel in case of side effects or other treatment concerns. Among the reasons for incomplete adherence recalled by providers, most incomplete adherence was due to the patient feeling better and deciding completion of the treatment regimen was unnecessary or forgetting to take medication when travelling to the forest.

### Adverse events

A total of six patients (0.63% of the 959 enrolled) with potential adverse events following radical cure treatment required referral to provincial hospitals throughout implementation. These patients were detected during HF follow up calls based on the paper registers collected by CSOs every month. Common side effects reported during HF follow up calls included nausea, shortness of breath, fatigue, back pain, and abdominal pain. Five of the six patients reached provincial hospitals and completed health assessments for adverse events. One patient refused to go to the hospital, citing mild symptoms and discontinued primaquine on the second day of treatment. Patients recovered quickly after stopping primaquine treatment as well as receiving medical treatment such as Intravenous therapy. All assessed adverse events were found not to be severe, requiring no additional intervention such as blood transfusion. Upon examination at provincial hospitals, none of the cases referred to provincial hospitals were found to be a result of primaquine administration.

### Change in *P. vivax* epidemiology

**Fig. 4:**
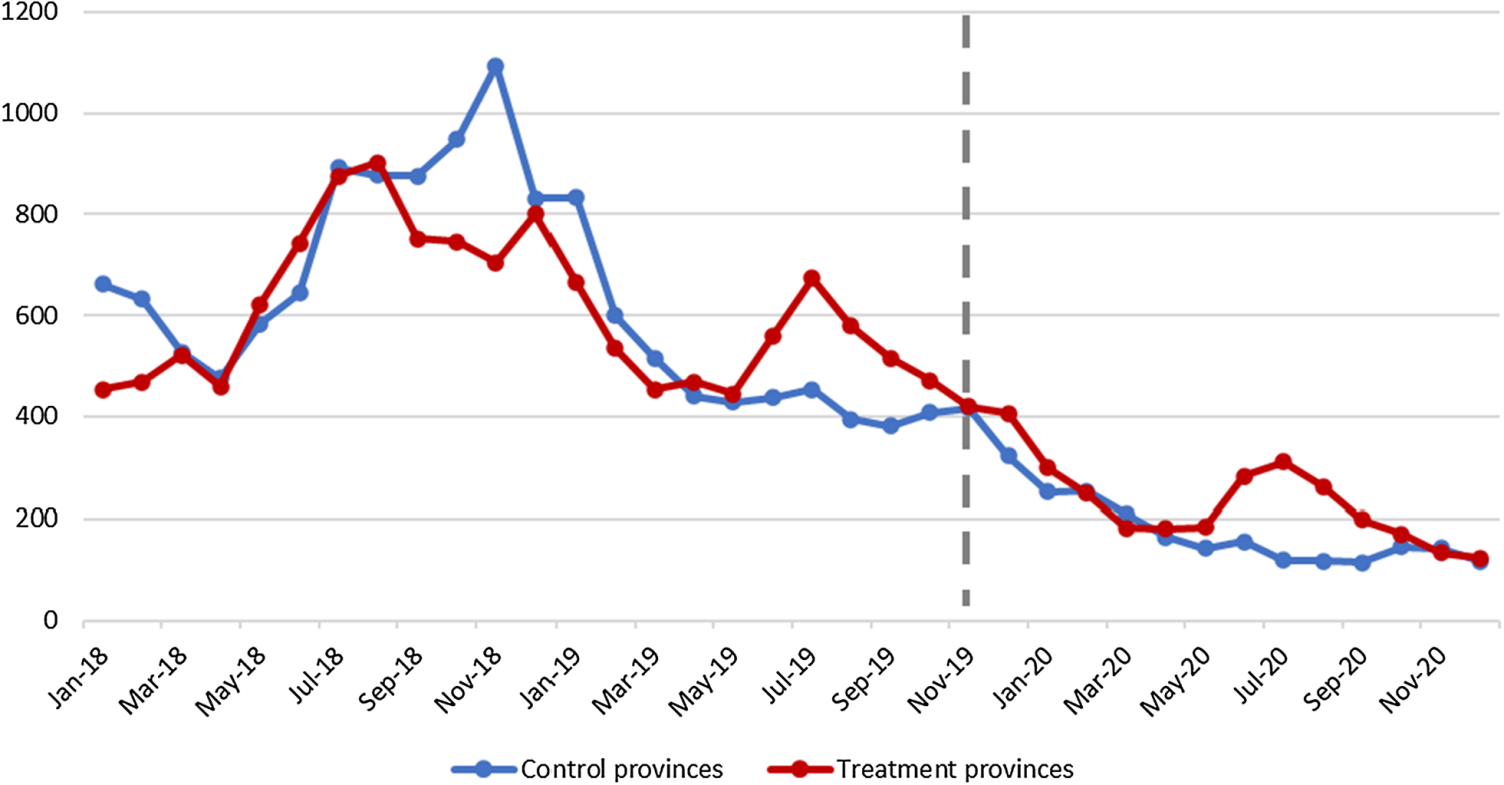
*P. vivax* cases in radical cure provinces, Jan 2018–Dec 2020. Dashed line indicates the month *of P. vivax* radical cure rollout

In January 2018, there were 3,146 *P. vivax* cases in Cambodia nationwide and 454 (14%) in the radical cure provinces. Two years later, in December 2020 after the Phase I implementation, there were 423 *P. vivax* cases nationwide and 123 (27%) in the radical cure provinces (Fig. 4). The national annual parasite incidence (API) of *P. vivax* declined from 5.02 in 2018, 3.01 in 2019 and to 0.90 in 2020, a decline of 82% throughout the three years. When stratified by implementation and non-implementation provinces, there is a 71% decrease in API in radical cure provinces, from 3.01 in 2018, 2.43 in 2019, and to 0.86 in 2020. While non-radical cure provinces declined 89% from 2.78 in 2018, 1.27 in 2019 and to 0.30 in 2020. This difference in API reduction may be due to the impact of Kampong Speu, one of the highest burden provinces in the country overall, which had the lowest radical cure coverage during phase I implementation and therefore made slower progress in reducing API during this period.

### Impact on *P. vivax* relapse rate

**Fig. 5:**
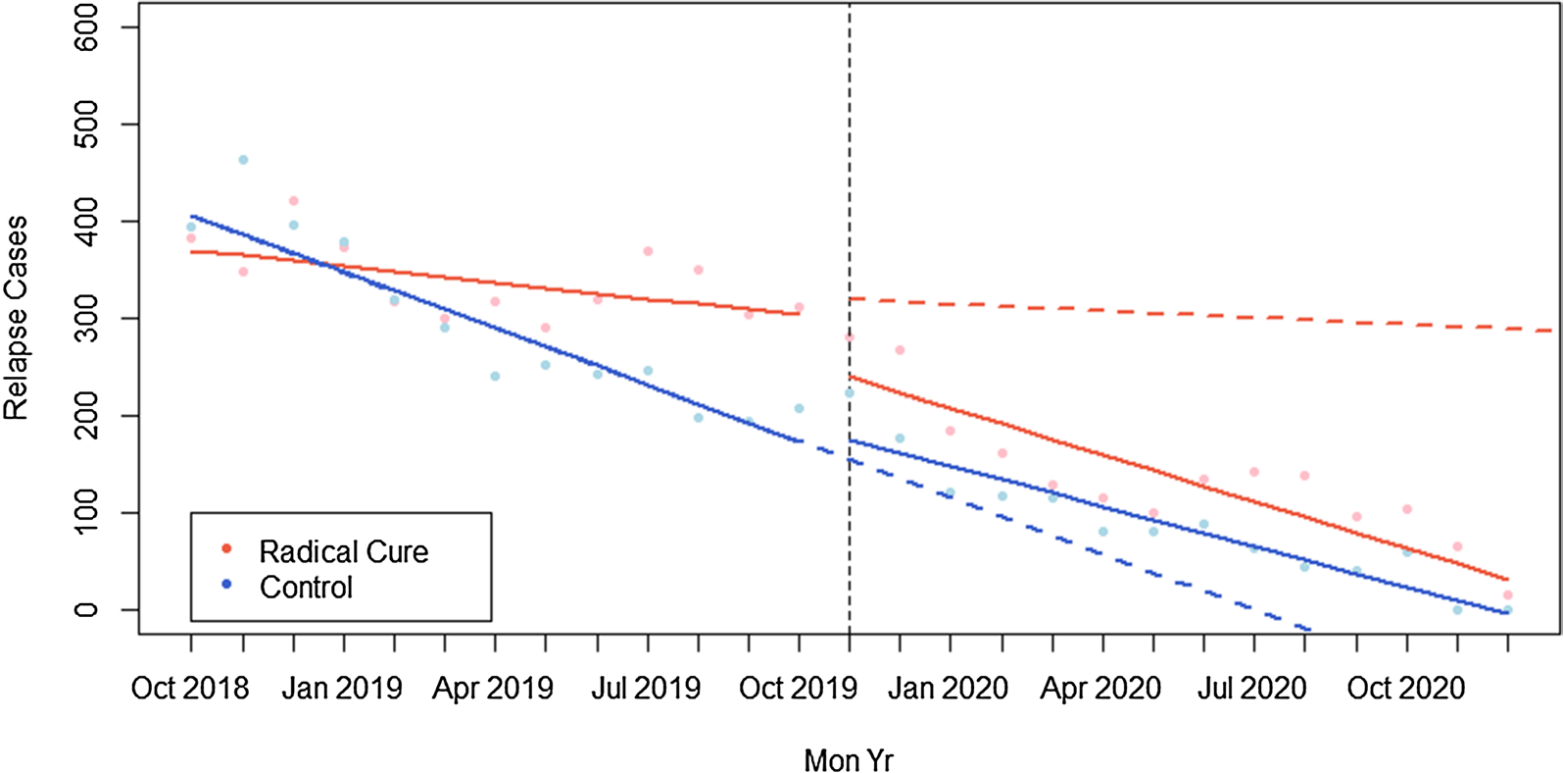
Number of relapse cases pre and post rollout of radical cure (October 2019) in intervention provinces (red) and control provinces (blue). Solid lines denote the linear regression model fit from the results. Dotted lines denote the counterfactual estimate for post-intervention extrapolated from the GLS regression model

From a total of 31,175 *P. vivax* patient records from October 2018 to December 2020 with complete identifying field information in the control or intervention provinces, 13,047 (41.8%) met the criteria for being a relapse case. At the beginning of the study period in October 2018, 394 cases in control provinces and 382 cases in intervention provinces were classified as relapses. Relapse incidence had decreased to 223 in control provinces and 281 in intervention provinces by November 2019 (start of radical cure) and to zero relapse cases in control provinces and 15 relapse cases in radical cure provinces by December 2020 (Fig. 5).

As mentioned previously, control provinces and intervention provinces did not have a similar *P. vivax* burden before phase I implementation. Prior to the intervention, relapse cases were already decreasing by an average of 19.28 per month in control provinces and increasing by an average of 13.75 per month in intervention provinces relative to controls because the intervention provinces represented a large burden of *P. vivax* in the country Post-intervention, from November 2019 to December 2020, relapse cases decreased by 15.8 (p < 0.001) cases per month in the intervention group, relative to the control group. In the 14th month after radical cure, the average monthly relapse cases were 286 fewer than would have been expected if radical cure had not been implemented, representing a 90% reduction in relapse cases relative to the counterfactual estimate for pilot provinces, accounting for level and trend changes in control provinces during the same period.

## Discussion

During the 14-month period in the four provinces, the coverage of safe radical cure was 29.6% among total *P. vivax* patients. Results demonstrated strengths in the feasibility of radical cure implementation at HF level, and its impact on reducing estimated relapse cases. However, challenges remain in the coverage and access of radical cure, the lack of validation for self-reported adherence, insufficient capacity and time of health centre staff in high-burden provinces, and a limited pharmacovigilance system.

Health centre staff were able to provide G6PD testing and 14-day primaquine to patients, follow up by phone call on day 3, 7 and 14 during the treatment, and record data on paper-based form. All patients received 14-day primaquine were tested G6PD normal, in accordance with the treatment guidelines, indicating the feasibility of radical cure implementation at HF level despite challenges of the new G6PD diagnostic tool and treatment regimen, and interpreting the result of G6PD qualitative tests based on colour grade instead of a control line. Over the course of phase I implementation, *P*. *vivax* cases decreased from 465 to 117 and an estimated 90% reduction in relapse cases compared to a counterfactual estimate. This reduction showed the potential impact of radical cure on reducing relapse. However, it is important to note that this effect could be underestimated given the short period of time of the data since the beginning of radical cure. Conversely tit could also be an overestimate if some cases were newly infected during the nine-month period. With the characteristic of relapse, *P. vivax* could cause other negative impacts on patients, including inpatient admission and economic losses, underscoring the importance of radical cure for malaria elimination [14]. Apart from its impact at the individual level, *P. vivax* infection also leads to additional case burden at the community level, as it can contribute to onward transmission from relapse cases.

Achieving high coverage of radical cure presents a challenge observed in Phase I. 71.4% of eligible *P. vivax* infections did not receive a G6PD test, mainly contributed by the low VMW/MMW referral completion rates. Since VMWs/MMWs were not able to provide G6PD testing and successful referral proved challenging in villages further away from HFs, this led to low treatment coverage. Among the non-successful referred patients, the majority were made up of mobile and migrant populations who work in forests and live further away from HFs. Given that the malaria burden primarily resides in hard-to-reach areas (e.g., forests), the availability of G6PD testing in those areas would be an issue given its infrastructural limitations [15, 16]. In addition, a study assessing 7 day PQ adherence in the Peruvian Amazon found that after patients completed ACT, malaria symptoms were often abated such that patients lacked motivation to travel for G6PD testing and further treatment. Similarly, for patients in areas with limited transport, adherence to medication decreased after day three [17].

In Cambodia, VMWs/MMWs play a crucial role in detecting malaria cases, conducting 73% of the total tests and detecting 60% of the total malaria cases in 2020. Therefore, successful utilization of these human resources for referral of *P. vivax* cases will be critical to the future success of radical cure. In addition to greater supervision and training for VMWs/MMWs and HFs [18], providing travel reimbursements to patients or financial incentives to VMW/MMWs for successful referral could strengthen the referral system. Another approach national programmes could take to increase coverage of radical cure is to conduct G6PD testing at the community or village level, removing the need for referral altogether. Concerns about the ability of VMWs to interpret G6PD results, lack of quality control, and insufficient storage capacities at the community level prevented CNM from implementing community level G6PD testing during phase I implementation. However, future development of G6PD tests that are easier to interpret or more durable to withstand field conditions, may encourage CNM or other malaria programmes to conduct community level G6PD testing.

In addition, lower coverage was observed in the higher burden HFs across multiple key indicators including VMW/MMW referral rate, G6PD testing, and treatment adherence compared to the other phase I provinces. This discrepancy might be due to the high caseload in the high-burden areas limiting HF and VMW availability. Thus, regular supervision of HFs, especially in high-burden areas, is necessary during implementation, to enhance the knowledge, practices, and quality of radical cure services. Furthermore, both VMWs and HF staff are responsible for other malaria activities outside of case management, such as case investigation, community mobilization and bed net distribution, which may interfere with their ability to provide high quality radical cure services. Lower testing performance in busier clinics and HFs has also been reported in other contexts, in which healthcare workers were unable to spend sufficient time with patients and therefore tests had poorer results [15].

Additional considerations the national programme must consider prior to nationwide scale up include that these results are not representative of the whole Cambodian population. Exclusion of female patients and patients under 20 kg due to limited G6PD qualitative tests and primaquine formulations, respectively, highlights significant coverage gaps that will need to be filled to reach elimination. Furthermore, regarding the adverse event reporting, only 6 patients with side effects were identified through the established adverse event reporting structure. However, key informant interviews at select health centres revealed at least 9 additional patients with side effects following primaquine treatment that were not captured in the adverse event forms. Thus, a more robust pharmacovigilance system including closer monitoring and supervision to hospitals and health centres as well as additional patient counselling resources to better identify and report adverse events is needed prior to nationwide scale up.

There are limitations concerning the available data and analysis. Patient data linkage in the interrupted time series analysis could be inaccurate if HF staff recorded patient data incorrectly. The relapse rate could be an overestimate as the chosen relapse timeline is selected from wider estimates reported in the literature. Conversely, given the lengthy relapse periods possible for *P. vivax*, these results may underestimate the total impact of radical cure on relapse cases in these provinces, as data reflect only 14 months following implementation.

Another limitation relates to the validity of treatment adherence data. According to self-reported adherence data, this Phase I implementation achieved at least 77.9% 14-day treatment completion. However, when this is compared to other studies, where the adherence rate for 14-day primaquine regimen ranged from 23.8% to 98% [19–21], Cambodia’s phase I implementation suggests higher compliance. This may be a result of overestimation of adherence by HF staff or patients either due to memory and/or social desirability biases [22]. Multiple approaches are recommended to follow up patients’ adherence, including leveraging cross-sectoral collaboration from healthcare professions, the community, and patients and their family [23]. Besides supervising adherence, health education via texts or other materials could improve compliance, even in the situation where healthcare workers are unable to reach the patients [19, 24].

## Conclusion

The strengths and challenges identified during Phase I have been used to inform the design and implementation strategy of the national level rollout. Since 2021, Cambodia began implementing *P. vivax* radical cure nationwide with the use of quantitative G6PD testing which allows for enrollment of both males and females. Furthermore, in August 2022 the country implemented a referral incentive programme for VMW/MMWs that accompany and provide patients with free transportation to HCs for *P. vivax* radical cure. With the goal to achieve malaria elimination by 2025, the national programme has further accelerated its *P. vivax* radical cure programme. Following updated WHO recommendations, CNM has transitioned from the 14-day PQ treatment to the shorter course 7-day PQ regimen (0.5 mg/kg per day) for G6PD normal patients and is planning to scale up an ongoing pilot for 8-week PQ (0.75 mg/kg/day) for G6PD deficient and intermediate patients.

### Supplementary Information

**Additional file 1: Table S1.** Primaquine dosing tables used for 7day Pv radical cure treatment 2019–2020. **Table S2.** Health facilities selected for key informant interviews. **Table S3.** Province Level *P. vivax* Incidence, 2019-2020. **Table S4.** GLS Regression Model (p=5).

## Data Availability

Data is available at National malaria surveillance data is available at https://mis.cnm.gov.kh/. Radical cure data is available upon reasonable request to the National Center for Parasitology, Entomology and Malaria Control (CNM), Phnom Penh, Cambodia.

## Abbreviations

ACT: Artemisinin-based combination therapy
ADR: Adverse Drug Reaction
AHA: Acute haemolytic anaemia
API: Annual parasite index
ASMQ: Artesunate-mefloquine
BMGF: Bill & Melinda Gates Foundation
CHAI: Clinton Health Access Initiative
CMEP: Cambodia Malaria Elimination Project
CNM: National Center for Parasitology Entomology and Malaria Control, Cambodia
CSO: Civil Society Organization
DDF: Department of Drugs and Food
ERP: External Review Panel
G6PD: Glucose-6-phosphate dehydrogenase deficiency
GF: Global fund
GLS: Generalized least squares
HC: Health centre
HF: Health facility
HSD: Health and Social Development Project
JW: Jaro-Winkler
MMV: Medicines for Malaria Venture
MMW: Mobile Malaria Worker
MOH: Ministry of Health
OD: Operational District
PAVE: The Partnership for Vivax Elimination
PH: Provincial hospital
PHD: Provincial Health Department
PSI: Population Services International
RH: Referral Hospital
UNOPS: United Nations Office for Project Services
VMW: Village Malaria Worker
WHO: World Health Organization
