# Supplementary material for: Radical cure for Plasmodium vivax malaria after G6PD qualitative testing in four provinces in Cambodia, results from Phase I implementation
Source: Malar J. 2024 Feb 23;23:56. doi: 10.1186/s12936-024-04884-4 (PMC10893713; doi:10.1186/s12936-024-04884-4)
Supplement: Supplementary file 1 — Additional file 1: Table S1. Primaquine dosing tables used for 7day Pv radical cure treatment 2019–2020. Table S2. Health facilities selected for key informant interviews. Table S3. Province Level P. vivax Incidence, 2019-2020. Table S4. GLS Regression Model (p=5). [file 12936_2024_4884_MOESM1_ESM.docx]

## Additional file 1: Captions

S1. Primaquine dosing tables used for 7day Pv radical cure treatment 2019 - 2020

| **Weight** | **For Administration at Public Health Facilities** | **# of days** |
| --- | --- | --- |
|  | **# of PQ Tablets** |  |
| **20kg - 30kg** | **1 tablet of 7.5mg** | **14** |
| **31kg - 45kg** | **1 tablet of 15mg**  (or 2 tablets of 7.5mg if 15mg unavailable) | **14** |
| **46kg - 60kg** | **1 tablet of 15mg + 1 tablet of 7.5mg**  (or 3 tablets of 7.5mg if 15mg unavailable) | **14** |
| **61kg – 99kg** | **2 tablets of 15mg**  (or 4 tablets of 7.5mg if 15mg unavailable) | **14** |

S2. Health facilities selected for key informant interviews

| **Province** | **OD** | **Facility** | ***P.vivax* cases (Nov 2019 – Oct 2020)** |
| --- | --- | --- | --- |
| Battambang | Maung Russei | Prey Tralach | 20 |
| Battambang | Sampov Luon | Trang | 24 |
| Battambang | Thma Koul | Khleang Meas | 6 |
| Battambang | Battambang | Ta Sanh | 1 |
| Kampong Chhnang | Kampong Chhnang | Akphivoadth | 54 |
| Kampong Chhnang | Kampong Chhnang | Chieb | 131 |
| Kampong Chhnang | Boribo | Trapeang Chan | 68 |
| Kampong Chhnang | Kampong Tralach | Thlok Vien | 4 |
| Kampong Speu | Kampong Speu | Trapeang Cho | 512 |
| Kampong Speu | Kampong Speu | Oral | 739 |
| Kampong Speu | Ou Dongk | Amleang | 7 |
| Kampong Speu | Phnom Srouch | Traeng Trayueng | 110 |
| Kampong Speu | Kong Pisey | Kak Preah Khe | 28 |
| Pailin | Pailin | Krachab | 6 |
| Pailin | Pailin | Phnom Spong | 2 |

S3. Province Level *P. vivax* Incidence, 2019-2020

| **Province** | **Radical Cure** | **PV Cases Oct 2019** | **Oct 2019 Inci-dence** | **PV Cases Feb 2020*** | **Feb 2020**  **Inci-dence** | **2019 PV Inci-dence** | **Mean LST Day 2018 (°C)** | **Population** | **Data Resolution** | **Inclusion Status** |
| --- | --- | --- | --- | --- | --- | --- | --- | --- | --- | --- |
| Kampong Chhnang | Rollout | 116 | 0.2 | 54 | 0.05 | 2.99 | 30.98 | 500,874 | Line list | Intervention |
| Battambang | Rollout | 34 | 0.03 | 11 | 0.01 | 0.4 | 31.23 | 1,351,868 | Line list | Intervention |
| Pailin | Rollout | 4 | 0.04 | 2 | 0.02 | 0.39 | 30.18 | 184,835 | Line list | Intervention |
| Kampong Speu | Rollout | 310 | 0.38 | 156 | 0.18 | 4.74 | 31.02 | 782,248 | Line list | Intervention |
| Kratie | No Inter-vention | 116 | 0.25 | 66 | 0.14 | 3.98 | 30.99 | 375,568 | Aggregate (HF) | Control |
| Siem Reap | No Inter-vention | 17 | 0.01 | 8 | 0.01 | 0.26 | 30.97 | 1,178,783 | Line list | Control |
| Kampong Thom | No Inter-vention | 21 | 0.03 | 17 | 0.02 | 0.63 | 30.63 | 673,506 | Line list | Control |
| Oddar Meanchey | No Inter-vention | 0 | 0 | 0 | 0 | 2 | 32.41 | 403,304 | Aggregate (HF) | Control |

S4. GLS Regression Model (p=5)


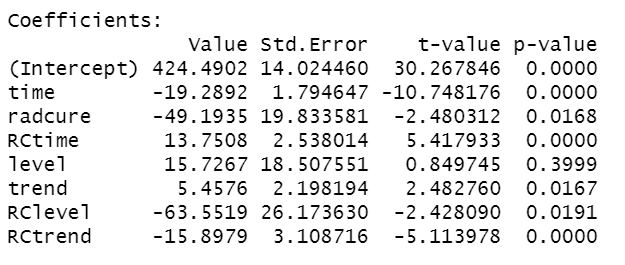


**Label name:**

- (intercept) level of relapse for controls at time 0
- time: existing trend in controls
- radcure: difference in level between radical cure & control
- RCtime: trend of radical cure over time relative to control
- level: after RC, increase in relapse in controls
- trend: after RC, increase in trend in relapse in controls
- RClevel: after RC, drop in relapse relative to controls
- RCtrend: after RC, drop in relapse trend relative to controls
